# From Therapeutic Drug Monitoring to Model-Informed Precision Dosing: A Review of Busulfan Dosing Optimization in Pediatric Hematopoietic Stem Cell Transplantation

**DOI:** 10.3390/pharmaceutics18091154

**Published:** 2026-09-15

**Authors:** Xiao-Ying Zhang, Yue Li, Jing Xu, Yong-Jun Fang, Xuan-Sheng Ding, Feng Chen

**Affiliations:** 1 Pharmaceutical Sciences Research Center, Department of Pharmacy, Children’s Hospital of Nanjing Medical University, 72 Guangzhou Road, Nanjing 210008, China; z1005990882@163.com (X.-Y.Z.); leey21012@163.com (Y.L.); njxujing@163.com (J.X.); 2 School of Basic Medicine and Clinical Pharmacy, China Pharmaceutical University, 639 Longmian Avenue, Jiangning District, Nanjing 211198, China; 3 Department of Hematology and Oncology, Children’s Hospital of Nanjing Medical University, 72 Guangzhou Road, Nanjing 210008, China; fyj322@189.cn

**Keywords:** busulfan, hematopoietic stem cell transplantation, pediatric patients, therapeutic drug monitoring, quantitative pharmacology, model-informed precision dosing

## Abstract

**Background/Objectives**: Busulfan is a cornerstone conditioning agent for pediatric hematopoietic stem cell transplantation (HSCT), yet its narrow therapeutic index and substantial pharmacokinetic (PK) variability complicate dosing. This review synthesizes current evidence on pediatric busulfan PK, exposure-guided dosing, and the transition from conventional therapeutic drug monitoring (TDM) to model-informed precision dosing (MIPD). **Methods**: We conducted a focused narrative search of PubMed, Web of Science, and ScienceDirect from database inception to August 2026 and synthesized relevant literature on pediatric busulfan PK, exposure–response relationships, TDM, population PK modeling, Bayesian methods, and emerging quantitative approaches. **Results**: Body size is the primary determinant of busulfan clearance in children, while maturation, underlying disease, concomitant medications, and pharmacogenetic variation may contribute additional variability, although their effects are inconsistent across populations. Busulfan exposure is associated with HSCT outcomes and toxicity; however, reported targets differ by dosing regimen, conditioning intensity, disease category, and exposure metric. Reliable exposure estimation requires accurate sampling records and validated bioanalysis, while limited sampling strategies may improve clinical feasibility. Integrating population PK models with Bayesian estimation enables individualized dose adjustment, although routine implementation requires external validation and compatibility with local workflows. Emerging approaches, including physiologically based PK, PK/PD, and machine learning models, show promise but lack sufficient clinical validation for routine use. **Conclusions**: TDM remains the foundation of pediatric busulfan precision dosing, while MIPD provides a framework for integrating patient characteristics, measured concentrations, and validated models. Standardized workflows, population-appropriate exposure targets, prospective validation, and evidence of improved clinical outcomes are essential for broader implementation.

## 1. Introduction

Hematopoietic stem cell transplantation (HSCT) is an important therapeutic option for a wide range of life-threatening malignant and non-malignant diseases in children, offering long-term survival and even cure in some cases [1]. As a critical component of HSCT, conditioning primarily aims to eradicate abnormal hematopoietic cells and residual disease, thereby creating a favorable environment for donor stem cell engraftment and proliferation. In addition, its immunosuppressive effects help reduce the risk of post-transplant rejection. Current international guidelines consistently emphasize that both the intensity and safety of conditioning are closely associated with transplant success and transplant-related mortality [2,3].

Conditioning regimens are currently classified according to intensity into myeloablative conditioning, reduced-intensity conditioning (RIC), and non-myeloablative conditioning [4]. The choice of regimen is influenced by multiple factors, including disease type, disease status, the patient’s clinical condition, and donor source [5]. Among the chemotherapeutic agents used in conditioning, busulfan (Bu) is widely regarded as a cornerstone drug because of its potent myeloablative activity and is recommended as a first-line component of multiple conditioning regimens in international guidelines. Bu-containing regimens therefore remain a mainstay of HSCT conditioning, and optimizing therapeutic management and risk monitoring during conditioning is essential for improving transplant safety and overall outcomes.

Bu is a cell cycle-nonspecific alkylating agent that exerts cytotoxic effects primarily by inducing DNA damage. Its clinical use is challenged by a narrow therapeutic window and substantial interindividual PK variability, and both efficacy and toxicity are strongly associated with the area under the concentration–time curve (AUC), indicating a clear exposure–response relationship. Accordingly, therapeutic drug monitoring (TDM) has become a routine supportive strategy in clinical Bu use. In recent years, with advances in quantitative pharmacology and model-informed precision dosing (MIPD), individualized Bu therapy has evolved from conventional dose-based administration toward a more dynamic and proactive precision dosing paradigm.

## 2. Literature Search

To inform this narrative review, a focused search of PubMed, Web of Science, and ScienceDirect was conducted from database inception to August 2026 using combinations of “busulfan”, “hematopoietic stem cell transplantation”, “pediatric”, “therapeutic drug monitoring”, “model-informed precision dosing”, “population pharmacokinetics”, “Bayesian”, “PBPK”, “PK/PD”, “machine learning”, “pharmacogenetics”, “glutathione S-transferase”, “GSTA1”, “bioanalytical methods”, “LC–MS/MS”, “HPLC”, and “gas chromatography”. Reference lists of key articles were also screened manually. The core scope focused on intravenous Bu used for conditioning in pediatric HSCT, including pharmacokinetics, exposure–response relationships, TDM, limited sampling, population PK modeling, Bayesian methods, and clinical implementation. Adult, oral-Bu, mechanistic, metabolite, and analytical studies were considered when they provided relevant historical or mechanistic context for pediatric intravenous Bu. Studies focused primarily on comparisons of conditioning regimens without an exposure or dosing component, non-HSCT indications, and formal quantitative evidence synthesis were outside the scope of this review. The earliest pediatric PK study retained for historical context was published in 1993, providing an evidence base spanning 1993 through August 2026.

## 3. Mechanism of Bu

**Figure 1:**
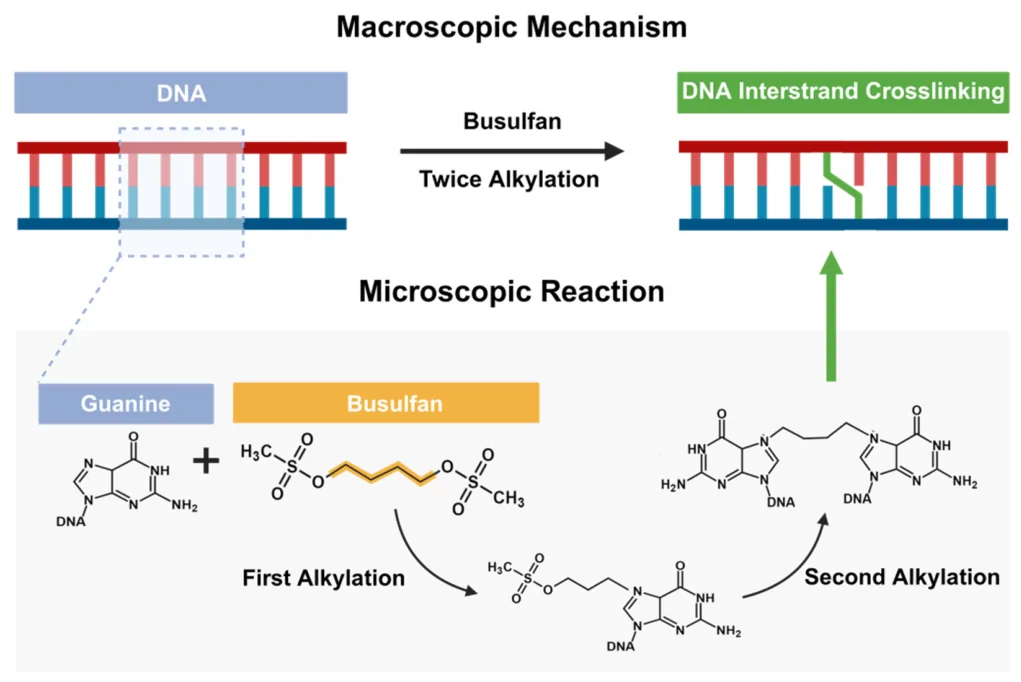
Mechanism of action of Bu. The schematic depicts the stepwise formation of DNA interstrand crosslinks by the bifunctional alkylating agent Bu. Bu sequentially alkylates the positions of two guanine residues. The initial monoalkylation is followed by a second alkylation event on the opposing DNA strand, culminating in a covalent interstrand crosslink that disrupts normal DNA structure and function.

Under physiological conditions, Bu first reacts with guanine residues in cellular DNA, releasing one methanesulfonate moiety and generating a monoalkylated intermediate. A second alkylation reaction then covalently links two guanine bases through the Bu tetramethylene chain, leading to the formation of DNA interstrand crosslinks and disruption of DNA structure and function (Figure 1). The myelosuppressive effect of Bu is mainly characterized by marked inhibition of granulopoiesis, whereas its suppressive effect on lymphocytes is relatively weak [6].

## 4. Significant Interindividual Differences in Clinical Efficacy and Safety

As the core component of conditioning regimens, the myeloablative intensity of Bu is directly associated with the therapeutic efficacy of HSCT. In myeloid malignancies, a clinical trial by Bredeson et al. showed that compared with total body irradiation (TBI), intravenous Bu was associated with better survival [7]. In lymphoblastic leukemia, Bu conditioning may be used as an alternative when TBI is contraindicated or unavailable [8]. Nevertheless, clinical practice has demonstrated that even under standardized dosing regimens, transplant outcomes remain highly unpredictable across pediatric patients.

### 4.1. Variability in Efficacy

Under standard weight-based dosing regimens, clinical responses to Bu are not uniform among pediatric patients. In children with hematologic malignancies, insufficient myeloablation is one of the key causes of early post-transplant relapse [9]. Bartelink et al. further demonstrated that under the same dosing regimen, the probability of achieving optimal therapeutic efficacy varied significantly among patients, with a markedly higher risk of relapse in the low-exposure group [10,11]. Ultimately, this instability in efficacy is reflected in differences in survival outcomes. Previous clinical data suggest that under standard dosing strategies, event-free survival (EFS) and OS remain suboptimal in a subset of pediatric patients [12].

### 4.2. Interindividual Variability in Safety

Interindividual variability in Bu-related toxicity represents another major clinical challenge during HSCT conditioning. Bu-related toxicities are highly heterogeneous and involve multiple organ systems, and their severity directly affects transplant-related mortality and long-term quality of life. Clinical studies have shown that Bu use is associated with a significantly increased incidence of complications such as oral mucositis, acute graft-versus-host disease (aGVHD), and hepatic veno-occlusive disease/sinusoidal obstruction syndrome (VOD/SOS) [10,13].

Among these, VOD is considered one of the most severe and potentially fatal complications of HSCT. Multiple studies have shown that the occurrence of VOD is closely associated with Bu use and systemic exposure, exhibiting a clear concentration-dependent pattern, with higher cumulative Bu exposure significantly increasing the risk of VOD [14,15,16,17]. Regarding central nervous system toxicity, previous studies have found that approximately 10% of patients receiving high-dose Bu may develop seizures; therefore, prophylactic anticonvulsant therapy has been widely adopted in clinical practice [18]. More recently, El-Serafi et al. reported that sulfolane, a major metabolite of Bu, can accumulate substantially in brain tissue and induce seizures as well as hypothermia, and that its plasma concentration may reflect actual central nervous system exposure [19]. In addition, studies suggest that higher drug exposure may aggravate mucosal barrier injury [20], while the incidence of aGVHD and non-infectious pulmonary complications also appears to be higher in Bu-based conditioning regimens and may be related to systemic exposure.

## 5. Marked Variability in the Disposition of Bu In Vivo

As a highly lipophilic small molecule alkylating agent, Bu exhibits substantial interindividual and intraindividual PK variability in children, which directly influences systemic exposure and represents a major determinant of variability in both efficacy and safety [21].

### 5.1. PK Profile of Bu

#### 5.1.1. Absorption and Distribution

Early oral Bu showed variable absorption, which contributed to the present preference for intravenous administration. After intravenous administration, Bu distributes rapidly into total body water and readily crosses the blood–brain barrier, with cerebrospinal-fluid concentrations approaching plasma concentrations. Reported binding is unusual for a reactive alkylating agent: approximately 32% is irreversibly associated with plasma proteins and approximately 47% with red blood cells, whereas the reversible protein-bound fraction is small [22]. These binding characteristics may be relevant when interpreting PK measurements obtained from plasma versus whole blood. However, the effects of hypoalbuminemia or changes in hematocrit on Bu disposition remain insufficiently characterized in pediatric patients. Extensive tissue distribution contributes to both myeloablative activity and neurotoxicity, including seizure risk [19,22].

#### 5.1.2. Metabolism and Excretion

Bu is primarily metabolized in the liver. Its core metabolic pathway involves either spontaneous conjugation or glutathione S-transferase (GST)-catalyzed conjugation with glutathione (GSH), leading to the formation of GSH–Bu conjugates [22]. Subsequent β-elimination generates tetrahydrothiophene (THT), which is sequentially oxidized to THT-1-oxide, sulfolane, and 3-hydroxysulfolane [22,23]. The parent drug mediates the established DNA-alkylating effects of Bu, whereas downstream metabolites have not been established as contributors to its conditioning efficacy. Nevertheless, selected metabolites may possess biological or toxicological activity. Experimental studies have shown that sulfolane can induce seizures and hypothermia, and it has also been detected in the plasma of patients receiving Bu. EdAG, an electrophilic metabolite generated through the GSH-dependent pathway, exhibits biochemical reactivity and cytotoxic potential in experimental systems [24]; however, its instability has precluded reliable direct quantification in vivo [23]. Thus, although downstream metabolites are not established mediators of the desired myeloablative effect, some may contribute to the biological or toxicological consequences of Bu metabolism.

**Figure 2:**
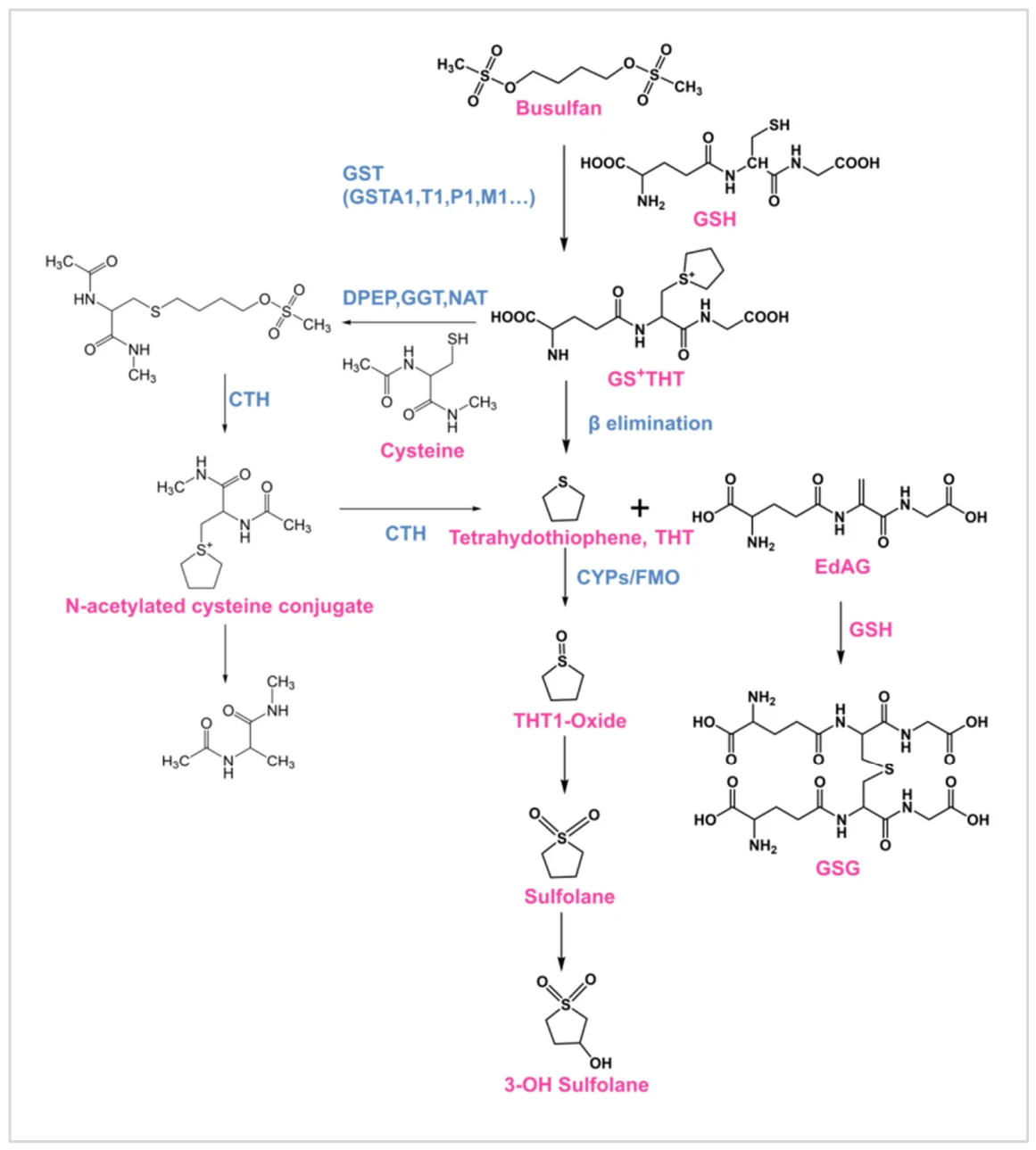
Hepatic metabolic pathway of Bu. Bu metabolism is predominantly driven by GST-mediated conjugation with GSH. This leads to a cascade of enzymatic reactions, most notably the formation of THT via beta-elimination. THT undergoes continuous oxidation by CYPs/FMO to form terminal circulating metabolites such as sulfolane and 3-OH sulfolane. Additional parallel processing routes resulting in cysteine conjugates and GSG are also depicted. The pathway was independently constructed by the authors using ChemDraw version 23.1.1 (32-bit) based on metabolic evidence synthesized from Myers et al. [22] and related literature.

The elimination half-life of Bu is approximately 2–3 h. Because Bu metabolism is accompanied by substantial depletion of intracellular GSH in hepatocytes, exhaustion of GSH stores may result in a time-dependent decline in Bu clearance (CL) [25]. The influence of polymorphisms in GST enzymes, which are the principal enzymes involved in Bu conjugation, on metabolic rate and interindividual exposure remains controversial. In addition, cytochrome P450 (CYP) enzymes and flavin-containing monooxygenases (FMOs) participate mainly in the downstream oxidation of THT and its metabolites rather than in the dominant initial clearance pathway of parent Bu [26,27] (Figure 2).

However, the contribution of membrane transporters to the disposition and elimination of Bu-derived metabolites remains incompletely characterized.

### 5.2. Variability in Bu Exposure

Because of the pronounced interindividual and intraindividual variability in Bu PK in children, systemic exposure may differ severalfold among patients receiving the same dose in the absence of individualized dose adjustment. Choong et al. reported a 4.2-fold interindividual variability in steady-state Bu concentration (C_ss_) after the first dose [28], while Kim et al. found that AUC could differ by more than two-fold among patients [29]. This variability is not limited to differences between individuals; substantial changes may also occur between dosing days within the same patient [30].

To better illustrate the extent of variability across studies, Table 1 summarizes key PK parameters reported in pediatric studies of intravenous Bu.

Because AUC has been reported in different units across pediatric Bu studies, direct comparison of exposure values may be difficult. To facilitate interpretation of Table 1, AUC values can be converted to a common unit using the molecular weight of Bu (246.3 g·mol^−1^). Specifically, AUC in mg·h·L^−1^ can be converted to μmol·min L^−1^ as follows:

$${\mathrm{AUC}}_{\mathrm{mg}\cdot h\cdot L^{-1}}\;=\;{\mathrm{AUC}}_{\mu \mathrm{mol}\cdot \min\cdot L^{-1}}\;\times \;246.3_{g\cdot {\mathrm{mol}}^{-1}}∕\left(1000\;\times \;60\right)$$

As shown in Table 1, published pediatric studies of intravenous Bu exhibit substantial heterogeneity not only in exposure estimates but also in study design and reporting practices. This cross-study variability makes direct comparison difficult and partly explains why fixed-dose nomograms alone are often insufficient in pediatric HSCT recipients. Taken together, these findings suggest that variability in Bu exposure is multifactorial, warranting further examination of physiological, pathological, genetic, developmental, and treatment-related determinants.

### 5.3. Factors Affecting Bu Exposure

#### 5.3.1. Physiological and Pathological Factors

As children grow and develop, physiological factors may substantially influence the disposition of drugs in vivo. Body weight is the major determinant of Bu CL, and pediatric dosing regimens are generally adjusted according to weight. Age may influence drug metabolism and elimination through enzyme maturation and changes in body composition. In addition to weight-based nomograms, body surface area (BSA)-based dosing adjustments are also used in several centers [48]. Meanwhile, a 5-year observational cohort found that sex had a significant effect on AUC [49].

In addition to physiological status, studies have shown that Bu CL may differ according to the underlying disease [50]. Bu CL appears to be slowest in children with immunodeficiency and fastest in those with hemoglobinopathies [51]. Another study further emphasized that the concentration of Bu and AUC in leukemia patients after the first dose were significantly higher than those in patients with non-malignant blood diseases [52]. These differences may be related to disease-associated microenvironmental changes, although the underlying mechanisms remain unclear. Evidence regarding the independent effects of hepatic dysfunction, malnutrition, and hypoalbuminemia on Bu clearance in pediatric patients also remains limited.

#### 5.3.2. Genetic Polymorphisms

Among the GST enzyme family, which plays a dominant role in Bu metabolism, multiple enzymes exhibit genetic polymorphisms, including promoter polymorphisms in alpha class enzymes such as GSTA1-1 and GSTA2-2, whole-gene deletions of *GSTM1* and *GSTT1*, and point mutations in *GSTP1* [53]. Among these enzymes, GSTA1 has the highest activity and is the principal enzyme responsible for Bu metabolism, whereas GSTM1, GSTT1, and GSTP1 appear to have weaker or synergistic effects [54]; the activities of GSTM1 and GSTP1 are estimated to be approximately 46% and 18% of that of GSTA1, respectively [55].

The *GSTA1* gene harbors functional single nucleotide polymorphisms mainly in its promoter region. Based on in vitro and hepatic tissue studies, Coles et al. showed that GSTA1 expression driven by the *A promoter is significantly higher than that driven by the *B promoter. Promoter polymorphisms therefore reduce GSTA1 expression and enzyme activity in carriers of the GSTA1*B allele [56]. By contrast, GSTA1*A/*A is generally associated with higher GSTA1 expression and activity, corresponding to faster metabolism, higher CL, and lower AUC. This effect appears to be particularly pronounced in children. Ansari et al. found that pediatric patients carrying the GSTA1*A haplotype had higher drug CL and lower drug concentrations [38]. However, large-scale whole-genome sequencing data from the Gnomad database indicate marked ethnic stratification in the allele frequency of the *GSTA1* rs3957357 variant [57]. In non-Finnish Europeans, the low-activity variant allele frequency is as high as 42.55%, whereas in East Asians it is much lower, at only 12.64%. The lower frequency of the low-activity variant therefore implies a lower proportion of genetically predicted slow metabolizers and, assuming a similar allele effect, a smaller population-level contribution of GSTA1 to Bu PK variability in East Asian cohorts, rather than absence of an effect in variant carriers.

In addition to GST enzymes, other drug-metabolizing enzymes may also affect Bu disposition. Uppugunduri et al. found that *CYP2C9* and *CYP2C19* polymorphisms were associated with THT metabolism and were further related to post-transplant overall survival and relapse rates [27].

#### 5.3.3. Ontogeny of Drug-Metabolizing Enzymes

At stages when enzyme expression has not yet fully matured, developmental factors may influence PK variability to an extent that they may modify or even mask the effects of genotype [58].

McCarver et al. reported that the expression and activity of some phase II drug-metabolizing enzymes, including GSTs, exhibit pronounced age dependence [59]. These developmental characteristics may mechanistically explain why Bu metabolic CL is relatively low in neonates and infants and increases substantially with age.

In addition to the main GST-mediated metabolic pathway, Bu metabolism also involves CYP-related enzymes such as CYP2C9 and CYP2C19, which participate mainly in the further conversion of intermediate metabolites. These enzymes likewise undergo age-dependent maturation, with expression and activity increasing progressively during development [60]. Although CYP-mediated pathways are not the dominant route of Bu metabolism, developmental differences in these enzymes may still affect metabolite elimination and, to some extent, overall exposure during specific developmental stages.

Taken together, in children, especially neonates and infants, Bu exposure is regulated not only by genetic factors but also by the developmental maturation of metabolic enzymes, This provides a plausible biological explanation for the heterogeneous responses to standard dosing observed in younger pediatric patients [61].

#### 5.3.4. Concomitant Medications

HSCT conditioning generally requires combination therapy together with supportive agents such as antimicrobials, hepatoprotective drugs, and anti-rejection medications to prevent serious adverse events. As a result, concomitant medication use in the clinical setting of Bu is highly complex.

Although prophylactic antimicrobial therapy as a whole has not consistently been associated with altered Bu PK in pediatric patients [62], drug-specific interactions have been reported. Itraconazole has been associated with reduced Bu clearance and increased exposure in some studies, whereas findings for other azole antifungals have been inconsistent. Acetaminophen has been proposed to interact with Bu through effects on GSH availability, although a consistently clinically relevant effect on intravenous Bu clearance has not been established.

Notably, many early PK studies of intravenous Bu were conducted in patients receiving phenytoin for seizure prophylaxis, which may have led to an underestimation of the actual Bu exposure achieved under intravenous infusion [63]. More recent studies support levetiracetam as a preferred alternative because of its lower potential for clinically relevant drug–drug interactions [64].

## 6. Exploration of Individualized Bu Dosing

In clinical practice, intravenous Bu is generally administered over four consecutive days, with either once-daily dosing or four-times-daily dosing. Although the four-times-daily dosing regimen remains the labeled schedule, once-daily dosing has been increasingly adopted because of its greater practical convenience and potential economic advantages [65]. Bazbaz et al. compared once-daily and four-times-daily intravenous Bu administration. Under MIPD-guided dosing, no significant differences were observed in EFS or safety, suggesting that dosing frequency itself may not be the key factor; rather, the critical issue is whether exposure-guided dosing can be achieved [66].

### 6.1. Clinical Value of TDM

At present, Bu clinical benefits have been widely demonstrated. Multiple studies have shown that traditional nomogram-based dosing based solely on body weight or BSA cannot accurately predict individual exposure or reliably achieve target exposure, and that individualized optimization through TDM is necessary to ensure the safety and efficacy of transplantation in pediatric patients [28,67].

A growing body of clinical evidence indicates that TDM can substantially improve Bu target attainment, thereby improving clinical outcomes and reducing adverse events. Hämmerle et al. compared pediatric patients receiving intravenous Bu with or without TDM-guided dose adjustment and found that the TDM group had significantly lower transplant-related mortality and significantly improved OS [68]. Similarly, in a study focused on VOD risk, Gokcebay et al. found that the incidence of VOD was significantly higher in the group without TDM intervention than in the TDM group [69]. Boelens et al. reported that after TDM intervention, the proportion of patients achieving the target Bu exposure increased from 49% to 87%, and the calculated number needed to treat was 2.5, indicating that TDM in 2.5 patients could prevent one additional adverse outcome [10]. In a cohort of pediatric patients, Shao et al. further demonstrated that TDM-guided individualized dose adjustment was associated with a high engraftment rate, a low incidence of severe toxicities, and favorable clinical outcomes [70]. Collectively, these findings consistently support the critical role of TDM in improving the prognosis of children undergoing HSCT.

### 6.2. Practical Considerations for Integrating Bu TDM and MIPD

Reliable implementation of Bu TDM and MIPD requires standardization across drug administration, sample collection, bioanalysis, exposure calculation and dose recommendation. In clinical practice, TDM and MIPD are complementary rather than separate processes: TDM provides patient-specific concentration data for exposure assessment, whereas MIPD integrates population PK information, patient covariates, and, when available, measured concentrations to support initial dose selection and Bayesian dose adjustment.

Infusion lag caused by tubing dead space or low infusion volumes may be particularly relevant in small children; actual infusion and sampling times should therefore be recorded, because unrecognized lag can bias AUC estimation, exposure classification, and subsequent dose adjustment [32,71]. Samples should preferably be obtained from a peripheral vein or a central-venous-catheter lumen not used for Bu infusion. Residual drug in the infusion lumen may produce spuriously high concentrations, overestimate exposure, and prompt inappropriate dose reduction [71].

#### 6.2.1. AUC Estimation and Limited Sampling Strategies

NCA, PopPK-based a priori prediction, and Bayesian estimation address different stages of exposure assessment. NCA numerically integrates the observed concentration–time profile without a population prior and generally requires sufficient samples to characterize the curve. A priori prediction uses population parameters and patient covariates before individual concentrations are available, whereas Bayesian estimation combines this prior with measured concentrations to estimate individual posterior PK parameters and exposure [72]. Bayesian methods can therefore support LSS. In an external evaluation of 11 published PopPK models, even the best model placed only 45.35% of a priori predictions within ±20% of observations; incorporating one to three concentrations improved individual prediction [73]. In addition, analysis of the same once-daily pediatric dataset yielded systematically higher cumulative AUC estimates with a model-based method than with NCA [74]. Exposure values and targets should therefore be interpreted in relation to the estimation method used.

Published pediatric LSSs indicate that sampling should be selected according to dosing schedule and body size. For q6h Bu infused over 2 h, two Bayesian samples obtained 0.25 and 4 h after the end of infusion reproduced NCA-based dose recommendations with 1.9% bias [75]. A single concentration obtained 6 h after infusion initiation (C6) performed better in children weighing >23 kg, whereas C3+C6 improved prediction in those weighing ≤23 kg [76]. Among the two- to four-point regression designs evaluated by Jia et al., sampling at 60, 135, 240, and 360 min from infusion initiation provided the highest accuracy [77]. Model-based optimization identified 0/3/4 h or 0/3 h after the end of infusion for q6h dosing, whereas q24h dosing favours later samples [78]. No single schedule has been validated across all pediatric age groups; q6h dosing also requires more rapid analytical turnaround than q24h dosing.

Day 1 is the practical minimum monitoring occasion because it permits early dose adjustment, but a fixed second monitoring occasion has not been established. In q6h-treated children, first-dose NCA and Bayesian estimates were both imprecise in predicting later exposure [79]. Bognàr et al. reported target attainment of 84.3% with Day 1 TDM and 90.9% with Days 1 and 2, without a statistically significant difference [80]. Under once-daily dosing, Lawson et al. observed a median 10.9% decrease in clearance from Day 1 to Day 4, whereas Puangpetch et al. found that adding Day 3 TDM narrowed the simulated exposure range [81,82]. The magnitude of this time-dependent change was smaller in some contemporary cohorts, possibly reflecting optimized conditioning and supportive regimens, although the mechanism remains uncertain [67]. Accordingly, repeat TDM should not be assigned routinely to Day 2, 3, or 4; the EBMT practical guide advises another TDM assessment on the following treatment day after a dose adjustment > 25%, if logistically feasible [71].

#### 6.2.2. Bioanalytical Methods and Quality Assurance

GC- and conventional HPLC–UV assays can reliably quantify Bu but commonly require extraction and derivatization [83,84]. LC–MS/MS methods can reduce specimen-volume requirements and analytical turnaround time [85]. In a direct comparison, HPLC–MS/MS provided faster sample preparation and showed close agreement with HPLC–UV for AUC estimation [86]. Regardless of the platform used, assay-specific validation and quality control remain essential. The bioanalytical method used in each pediatric study is summarized in Table 1.

International proficiency testing demonstrates that variability occurs across centers and may persist within a center over time. In the first two BuQMD rounds, 67–85% of reported concentrations were within ±15% of reference values, while 71–88% of dose recommendations met the predefined accuracy limits [87]. In subsequent rounds, approximately 75–80% of AUC calculations, but only 60–69% of dose recommendations, were considered accurate, and some laboratories showed repeated concentration deviations > 15% [88]. Quality assurance should therefore encompass concentration measurement, AUC calculation, and translation into a dose recommendation, with ongoing internal quality control and periodic external proficiency assessment rather than initial assay validation alone.

#### 6.2.3. Minimum Operational Requirements

A safe Bu TDM service requires a written administration and sampling protocol with exact-time capture; a validated plasma assay with internal quality control and, where available, external proficiency testing; trained clinical, laboratory, and pharmacy staff; and a validated pathway for exposure calculation and dose recommendation. To minimize infusion lag, the administration protocol should standardize tubing configuration and dead-space management, including line priming and post-infusion flushing procedures, and should require documentation of the actual infusion start and end times rather than scheduled times alone. The calculation method, model and software version, dosing schedule, units, and target definition should be controlled, with independent verification and reporting before the next actionable dose. Centers without an in-house pharmacometrician may use validated external analytical or Bayesian support, provided that local responsibility for data quality, clinical interpretation, and dose authorization is maintained [71].

### 6.3. Defining the Target Exposure Range

Defining an appropriate Bu target exposure requires balancing conditioning efficacy against treatment-related toxicity. The resulting therapeutic window is influenced by the dosing schedule, exposure metric, conditioning intensity, underlying disease and therapeutic objective, and the method used to estimate exposure. Per-dose or interval AUC, cumulative AUC, C_ss,_ and C_max_ represent distinct exposure metrics, whereas NCA, Bayesian estimation, and PopPK/NLME approaches describe how these metrics are estimated. The principal exposure targets and exposure–outcome thresholds reported in pediatric HSCT are summarized in Table 2.

#### 6.3.1. Exposure–Outcome Evidence Defining the Therapeutic Window

Clinical studies broadly support an exposure–response relationship for Bu. Lower exposure has been associated with graft failure, relapse, and inferior EFS, whereas higher exposure has been associated with increased treatment-related toxicity. An early pediatric study identified a cumulative exposure of approximately 78 mg·h·L^−1^ as being associated with favorable outcomes [11]. A subsequent multicenter analysis of 674 children and young adults identified a broader cumulative AUC of 78–101 mg·h·L^−1^ as the range associated with the best balance between EFS and toxicity [10]. A pediatric meta-analysis similarly found an increased risk of graft failure with lower exposure [97]. Using first-dose exposure rather than cumulative AUC, Ansari et al. reported that a first-dose C_ss_ > 600 ng·mL^−1^ was associated with higher NRM and grade II–IV aGVHD, and with lower EFS and OS [96]. In a later analysis from the same center focusing on children with AML or MDS undergoing unrelated UCBT, Benadiba et al. found that first-dose C_ss_ > 600 ng·mL^−1^ was associated with poorer neutrophil and platelet recovery, higher NRM, and lower EFS and OS [34]. More recently, in pediatric patients with ALL undergoing HLA-matched allogeneic HSCT with fludarabine–busulfan–thiotepa conditioning, a cumulative AUC of 73.3–98.0 mg·h·L^−1^ was associated with favorable EFS and GVHD-free, relapse-free survival, with relapse predominating at lower exposure and toxicity or GVHD at higher exposure [94].

The upper exposure boundary varies according to the toxicity endpoint and the way exposure is characterized. Higher mean AUC has been associated with VOD/SOS [97], whereas Philippe et al. identified the maximum estimated Bu concentration over the entire treatment course as a strong predictor of VOD [16]. Bognár et al. found an association between cumulative AUC > 78 mg·h·L^−1^ and VOD/SOS when Bu was the only alkylating agent, highlighting the influence of conditioning partners on this relationship [17]. Neurological complications have also been associated with AUC_96_ > 80 mg·h·L^−1^ [93]. In a recent study using once-daily Bu, higher end-of-infusion C_max_ was associated with liver-function-test elevation and poorer OS [95].

Collectively, these findings support a narrow Bu exposure–response window while showing that the apparent toxicity boundary varies across exposure metrics, conditioning regimens and clinical endpoints [10,97].

#### 6.3.2. Regulatory and Consensus Exposure Targets

Regulatory and consensus targets provide clinically established reference ranges but are specific to their original dosing schedules and exposure metrics. For q6h intravenous Bu, the FDA label specifies a per-dose AUC of 3.69–5.54 mg·h·L^−1^, with a desired exposure of 4.62 mg·h·L^−1^, whereas the EMA product information gives a broader range of 3.69–6.16 mg·h·L^−1^ per administration. For cumulative exposure, the 2024 EBMT practical guide highlights approximately 78–101 mg·h·L^−1^ as the best-supported range for pediatric myeloablative conditioning [10,71]. Lower cumulative exposure, including approximately 60–70 mg·h·L^−1^, is used in selected reduced-intensity protocols for inborn errors [71]. These reference ranges therefore reflect both dosing schedule and conditioning intensity.

#### 6.3.3. Population- and Disease-Specific Interpretation of Exposure Targets

More recent studies have refined cumulative exposure ranges for selected pediatric populations. In 57 patients with non-malignant disorders, Apsel Winger et al. proposed a target of approximately 75 mg·h·L^−1^, with a range of 70–80 mg·h·L^−1^, based primarily on sustained high-level donor myeloid chimerism and disease correction [91]. In a multicenter cohort of 562 patients with IEI, Bognár et al. identified approximately 80 mg·h·L^−1^, with a range of 70–90 mg·h·L^−1^, as a favorable exposure range. Donor chimerism increased with exposure and approached a plateau at approximately 90 mg·h·L^−1^, although the exposure–EFS relationship differed across IEI subgroups [92].

These population-specific ranges overlap the previously reported cumulative range of 78–101 mg·h·L^−1^. The 70–90 mg·h·L^−1^ range overlaps it substantially, whereas 70–80 mg·h·L^−1^ is narrower and shifted toward lower exposure. The pediatric ALL range described above also overlaps numerically with both ranges. This pattern may reflect the conditioning objectives of many non-malignant diseases and IEI, in which durable engraftment and sufficient donor chimerism for disease, metabolic or immune correction may be more relevant than maximal eradication of recipient hematopoiesis. Differences in disease composition, conditioning intensity, donor source, co-conditioning agents, exposure estimation, and endpoint definition may also contribute to the observed exposure–outcome relationships.

Population- and regimen-specific evidence has also emerged from Chinese pediatric cohorts. Du et al. reported better EFS with a model-derived per-dose AUC of 3.90–6.57 mg·h·L^−1^ in children receiving a 16-dose q6h regimen [89]. In a larger multicenter cohort, a model-derived first-dose AUC ≥ 4.5 mg·h·L^−1^ was associated with higher 2-year EFS. Cumulative exposure thresholds differed according to treatment duration, with AUC_cum_ ≥ 54 mg·h·L^−1^ in the three-day regimen and 70–90 mg·h·L^−1^ in the four-day regimen associated with higher 2-year EFS [90]. These findings further illustrate that favorable exposure thresholds and ranges vary with treatment schedule and population while remaining broadly consistent with the established exposure–response framework. Importantly, numerical exposure targets should not be compared directly across studies when different exposure-estimation approaches are used, particularly NCA and model-based methods.

### 6.4. Quantitative Pharmacology Tools Supporting MIPD

To overcome the limitation of traditional TDM, which can only guide dose adjustment after drug administration, quantitative pharmacology model-based approaches are increasingly being used as key tools to optimize the initial Bu dose.

#### 6.4.1. Population Pharmacokinetic (PopPK) Models

PopPK models quantify fixed and random effects in population data and identify key covariates affecting Bu disposition. Several systematic reviews have compared the determinants of Bu CL and the optimal target exposure ranges reported in PopPK studies [97,98,99,100]. A large body of evidence confirms that body weight is the most important determinant of Bu CL. In addition, some PopPK models have used body surface area (BSA) as the primary covariate and proposed corresponding dosing regimens [101,102,103,104], while other studies have explored the influence of GST activity on Bu CL [25,105]. The major covariate domains incorporated in pediatric i.v. Bu PopPK models are summarized in Table 3.

In recent years, model development has increasingly incorporated genetic factors. Several studies have shown that including GSTA1 polymorphisms can significantly improve the predictive performance of models for Bu clearance. In the model established by Nava et al., GSTA1 genotype was identified as a significant covariate, with carriers of loss-of-function alleles exhibiting lower CL [106]. Johnson et al. found that children carrying GSTA1*B had AUC values up to 2.6-fold higher than those of non-carriers, confirming the substantial impact of this genotype in pediatric populations [54]. Evidence from East Asian cohorts suggests that GSTA1 can influence Bu clearance, but its incremental value for initial-dose prediction is variable. In Chinese children, Yuan et al. retained GSTA1 as a clearance covariate, with 17.3% lower clearance in *B carriers, although the *B haplotype frequency was only 11.9% [101]; neither GSTA1 nor GSTP1 was significant in the model of Du et al. [89]. In the larger analysis by Huang et al., slow GSTA1 metabolizers had 13.5% lower clearance, with the reduction in OFV limited [90]. Puangpetch et al. screened GSTA1, GSTP1, and GSTM1 in Thai children and retained only GSTA1, which was associated with lower clearance in variant carriers; however, its inclusion produced little improvement in predicted exposure variability [82]. Yin et al. also detected genotype–PK associations despite the absence of GSTA1 *B/*B homozygotes [107], whereas Sun et al. found only a small reduction in between-subject clearance variability after inclusion of GSTA1 [108]. Collectively, these findings suggest that the relatively low frequency of GSTA1*B may limit its population-level contribution and the incremental value of genotype-informed initial dosing, without excluding a PK effect in variant carriers. This pattern does not imply an alternative metabolic pathway. Genotype may refine the population prior, whereas concentration-guided Bayesian estimation remains central to individual dose adjustment.

Random-effects components require separate interpretation. Interindividual variability (IIV) describes between-patient heterogeneity in PK parameters after accounting for fixed covariate effects and is routinely estimated for CL and usually for one or more distribution parameters. Intraindividual variability is broader: some models represent a deterministic treatment-day or continuous-time effect on CL, whereas inter-occasion variability (IOV; also termed between-occasion variability) represents random deviations in PK parameters between predefined doses or treatment days. These components may coexist and should not be interpreted as interchangeable. IOV is not consistently included because its estimation requires repeated PK observations across clearly defined occasions. When reported, CL IOV estimates are generally in the low-double-digit range, although larger values have been observed in some models. Potential contributors include evolving organ function or inflammatory status, time-varying co-medications, treatment-course physiology, and occasion-specific administration conditions. Residual unexplained variability (RUV) is the observation-level variability remaining after the structural model, covariates, IIV, and any IOV have been considered. Comparable proportional RUV terms range from low single-digit values to more than 20% across reported pediatric models. RUV may reflect assay error, sample contamination, discrepancies between actual and recorded infusion or sampling times, handling of below-quantification-limit observations, structural misspecification, and unmeasured covariates. Measurement and recording errors therefore primarily contribute to RUV, although errors clustered by occasion may be absorbed into an IOV estimate if they are not measured or modeled explicitly.

Beyond covariate identification, PopPK models should undergo external validation in independent cohorts and within the intended clinical setting before being applied to dose individualization, in order to establish their applicability across different ethnic backgrounds, dosing schedules, sampling designs, and analytical platforms. Systematic external evaluations of published Bu PopPK models have consistently demonstrated that only a limited number of models provide satisfactory a priori predictive performance, whereas the inclusion of one or a few measured concentrations can substantially improve the accuracy and precision of Bayesian estimation [73]. Earlier multicenter external validation studies in pediatric populations likewise suggested that only models validated across centers are more likely to provide robust support for individualized dosing [109]. In addition, whether a model can be successfully translated into a routine clinical tool also depends on its compatibility with the local TDM workflow.

MIPD combined with Bayesian methods is currently regarded as a preferred approach for individualized Bu dosing [98]. Before treatment, an initial dose is selected using a population PK (PopPK) model together with available covariates. After the first dose, a certain number of timed plasma samples are collected and entered into a Bayesian estimation framework. In this process, the PopPK model serves as the prior information, while the measured concentrations are used to update the individual estimates of CL and distribution, yielding posterior PK parameters. These posterior estimates can then be used to calculate the patient’s actual exposure and to simulate alternative doses for subsequent administrations to reach the predefined target AUC. When target attainment remains uncertain, repeat sampling and model updating may be performed during the remaining doses. This strategy allows early and individualized dose adjustment while reducing sampling burden, which is particularly advantageous in pediatric HSCT.

Hassine et al. demonstrated that MIPD-based dose adjustment effectively achieved target exposure in both adults and children and outperformed traditional empirical dosing [110]. Once a model has been adequately validated, embedding the MIPD algorithm into dose-decision support software enables standardized translation of model outputs into clinical dose adjustment, thereby greatly improving the practical feasibility of this strategy in routine care [111].

**Figure 3:**
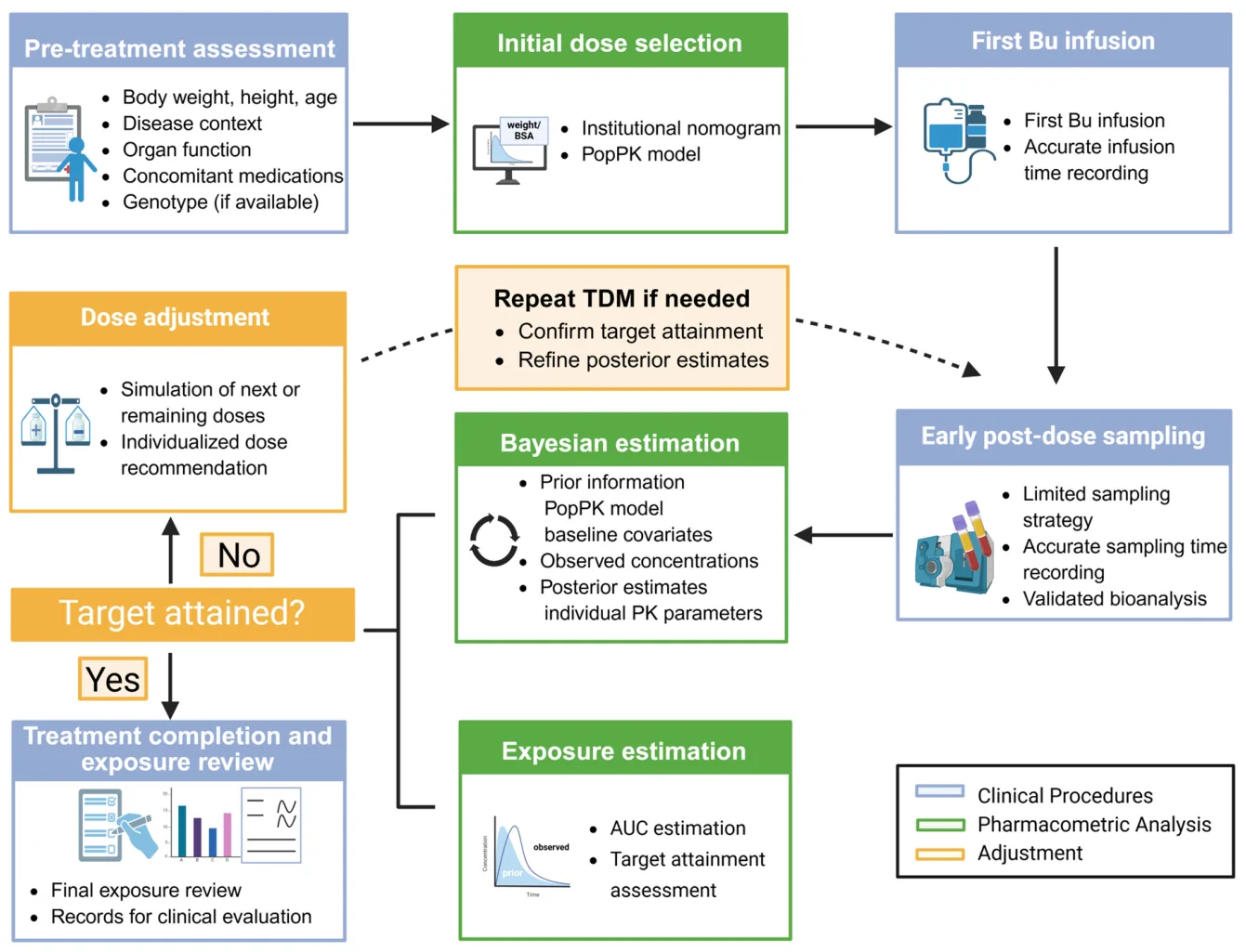
Proposed clinical workflow for MIPD of Bu in pediatric HSCT. Baseline covariates available before treatment, including body weight, age, disease context, organ function, concomitant medications, and pharmacogenetic information when available, are used to inform initial dose selection. After the first dose, limited post-dose blood samples are collected and entered into a Bayesian estimation framework. Within this Bayesian workflow, prior information (population PK model and baseline covariates) is combined with observed concentrations to obtain posterior estimates of individual pharmacokinetic parameters and achieved exposure. These posterior estimates are then used to estimate AUC and guide adjustment of subsequent doses toward the predefined target range. Repeating TDM may be performed when target attainment remains uncertain. Standardized sampling procedures, validated bioanalysis, and multidisciplinary coordination are essential throughout the workflow.

As illustrated in Figure 3, we proposed a practical MIPD workflow for Bu administration. In this framework, model-based initial dose selection is followed by early limited sampling and Bayesian estimation after the first dose, allowing posterior PK parameters to guide subsequent dose adjustment. The success of this workflow depends on model performance, accurate infusion and sampling time records, validated bioanalysis, and close coordination between clinicians, pharmacists, and the laboratory.

**Table 3:** Major covariate domains incorporated in i.v. Bu PopPK models for pediatric HSCT patients.

Covariate Domain	Common Representation	Main PK Parameter	Pattern Across Pediatric PopPK Models	Clinical Interpretation
Actual or total body weight	ABW/TBW, per-kilogram normalization, or weight-based linear or power functions	CL and V; occasionally other distribution parameters	Widely used and the most consistently incorporated primary size descriptor	Readily available for initial-dose calculation
Fat-free mass or normal fat mass	FFM- or NFM-based allometric scaling	CL and V	Incorporated in several models, dependent on the population and equation used	Explicitly represents lean and fat-mass contributions
Body surface area	Normalized BSA or BSA-based power functions	CL and V	Incorporated in selected models	Provides an alternative size descriptor
Allometric or empirical size scaling	Fixed or estimated power exponents, including a fixed CL exponent of 0.75; piecewise or age-dependent functions	Primarily CL and V; sometimes Q	Commonly used, the functional form and estimated exponents vary	Accounts for nonlinear relationships between body size and CL/V across growth
Age and maturation	Postnatal age, postmenstrual age, age groups or cutoffs, maturation functions, or age-dependent size exponents	Primarily CL; occasionally V	Retained in several models, particularly those including infants and young children	Captures developmental changes not fully explained by body size and is most relevant during early life
Treatment course or time	Treatment-day indicators or continuous time functions	CL	Incorporated in selected models not consistently included	Represents a systematic within-course change in CL
GST-related factors	GSTA1 genotype or diplotype, GST activity, or a GST/GSH-related semi-mechanistic term	CL or a parameter governing GSH-dependent elimination	Retained in selected models	May refine the population prior in some settings
Other clinical and treatment factors	Liver-function markers such as AST, disease category, conditioning regimen or concomitant medication, and sex	Mainly CL; occasionally V	Usually isolated or cohort-specific findings	Should be verified and applied carefully in combination with a specific cohort

Abbreviations: ABW, actual body weight; AST, aspartate aminotransferase; BSA, body surface area; CL, clearance; FFM, fat-free mass; GSH, glutathione; GST, glutathione S-transferase; IOV, inter-occasion variability; NFM, normal fat mass; Q, intercompartmental clearance; TBW, total body weight; V, volume of distribution.

As a simple clinical illustration, consider a 10 kg child receiving intravenous busulfan every 6 h. Based on the model-informed regimen reported by Neely et al., the initial dose would be 1.1 mg·kg^−1^ (11 mg) infused over 2 h, with a target average concentration of 600–900 ng·mL^−1^, corresponding to an AUC_0–6_ of approximately 3.6–5.4 mg·h·L^−1^. After the first dose, measured concentrations can be incorporated into the population PK model through Bayesian estimation to obtain patient-specific posterior PK estimates. For illustration, if the resulting posterior clearance were 3.2 L·h^−1^, the estimated first-dose AUC_0–6_ would be 11/3.2 = 3.44 mg·h·L^−1^, corresponding to an average concentration of approximately 573 ng·mL^−1^, indicating exposure below the target range. Taking the midpoint of the reported target range (AUC_0–6_ = 4.5 mg·h·L^−1^) as the desired exposure, the posterior clearance would correspond to a subsequent dose of approximately 4.5 × 3.2 = 14.4 mg. Thus, measured concentrations update the population-based prior estimate of pharmacokinetics and translate directly into an individualized dose for the next administration [75].

#### 6.4.2. Physiologically Based Pharmacokinetic (PBPK) and Pharmacokinetic/Pharmacodynamic Models

Unlike primarily empirical PopPK models, PBPK models represent drug disposition using physiological, anatomical, and drug-specific mechanistic information and can therefore support extrapolation across pediatric age groups. In an early pediatric Bu PBPK study, an adult model was scaled to children by incorporating GST expression across multiple organs and an age-dependent maturation function for Bu–glutathione conjugation. The model adequately described an external pediatric dataset and was proposed to support first-dose selection [112]. More recently, PBPK modeling in the HSCT setting incorporated age-dependent enzyme activity to simulate Bu exposure in children and was used to explore the effect of phenytoin co-administration and alternative Bu doses, illustrating its potential for mechanistic assessment of drug interactions and prospective dosing scenarios [113]. However, disease-specific physiological changes associated with HSCT have not yet been adequately incorporated into Bu PBPK models.

Mechanistic PK/PD models linking exposure to neutrophil kinetics may also support schedule- or growth factor–related questions, although restrictions still exist [114,115].

#### 6.4.3. Machine Learning (ML) Models

With the expansion of medical big data, ML has emerged as a powerful analytical tool and has, therefore, been increasingly applied to TDM and individualized therapy.

Comparative studies between ML and traditional PopPK models have found that ML may provide better goodness-of-fit and higher accuracy in predicting Bu AUC [116]. In recent years, the application of ML has expanded from concentration prediction alone to early warning of clinical toxicity. Using ML combined with explainable analysis, Lee et al. highlighted the importance of adherence to recommended dosing in preventing VOD [117]. These developments indicate that individualized Bu therapy is evolving beyond PK alone toward a precision medicine framework integrating multi-omics data.

## 7. Conclusions and Perspectives

As a cornerstone agent in pediatric HSCT conditioning, the clinical use of Bu has long been challenged by a narrow therapeutic window and substantial interindividual and intraindividual PK variability. Under conventional dosing strategies, this variability exposes pediatric patients to dual risks: insufficient myeloablation leading to relapse or graft failure, and excessive exposure leading to severe toxicity. Together, these risks can substantially compromise transplant success and long-term survival. Although TDM has been established in international guidelines as a key strategy for optimizing Bu exposure and improving clinical outcomes, its routine implementation in pediatric practice remains limited. Moreover, currently recommended exposure targets are still supported by insufficient multi-ethnic data and may not fully account for the diverse physiological, pathological, and genetic characteristics of individual pediatric patients.

To achieve truly safe and effective individualized Bu therapy in pediatric HSCT, coordinated efforts are urgently needed in the following areas:

### 7.1. Establish a Robust Evidence Base Across Diverse Pediatric Populations

High-quality, multicenter, large-scale, prospective clinical studies and real-world studies are needed. Such research should systematically collect comprehensive information in order to define and validate the optimal Bu exposure windows for different disease categories and conditioning intensities.

### 7.2. Develop and Validate Advanced Quantitative Pharmacology Models

Future studies should re-evaluate established covariates using complementary approaches, including mechanistic PBPK modeling, machine learning, and hybrid PopPK–machine learning frameworks, which may better characterize nonlinear relationships and interactions among patient factors. Candidate predictors should also extend beyond conventional clinical and pharmacogenetic variables to multi-omics data, such as metabolomic and lipidomic profiles, as well as dynamic biomarkers related to metabolism, inflammation, and treatment response. Prospective multicenter studies with longitudinal PK sampling and synchronized collection of clinical and biological variables are needed to evaluate these factors and determine whether they provide reproducible improvements in exposure prediction and individualized dosing.

### 7.3. Promote the Clinical Implementation of MIPD

Fully validated quantitative pharmacology models should be integrated with Bayesian methods to establish standardized MIPD workflows.

### 7.4. Build Standardized TDM Workflows and Data-Sharing Platforms

Pediatric HSCT centers should establish unified and standardized Bu TDM procedures, quality-control systems, and data-sharing platforms to support continuous model refinement and broader implementation of individualized dosing strategies.

## Figures and Tables

**Table 1:** PK parameters reported for i.v. Bu in pediatric patients.

Ref. *	Country	Age (Years)	Weight (kg)	Bu Dose (Times; Days)	TDM Schedule (Dose No.)	Sampling Time (h)	CL (L·h^−1^·kg^−1^)	V (L·kg^−1^)	Exposure Metric AUC (mg·h·L^−1^)	Bioanalytical Method
[31]	Japan	7.6 [0.4–11.8]	17.8 [6.5–46.3]	QID/QD; 4	QD/QID 1	TD: 0.08, 1, 2, 4, 8; RD: 0.08, 1, 2, 4	TD: QID 0.217 ± 0.0732; QD 0.187 ± 0.0667; RD: QID 0.254 ± 0.104; QD 0.33 ± 0.133	TD: QID 0.78 ± 0.15; QD 0.73 ± 0.26 RD: QID 1.03 ± 0.21; QD 1.71 ± 0.54	TD: QID AUC_6_: 2.64 ± 0.25; QD AUC_6_: 3.01 ± 0.31 RD: QID AUC_cum_: 63.20 ± 8.68; QD AUC_cum_: 38.30 ± 7.70.	LC–MS/MS
[32]	Greece	6.5 [0.5–19]	26 [7.38–93]	QID; 4	1	0, 2, 2.5, 4, 6	0.27 [CV 33.3%]	NR	AUC_6_: 3.95 [3.38–5.70] (CV 33.1%)	HPLC–PDA
[33]	Japan	8 [0.5–17]	NR	QID; 4	1	2, 3, 4, 6	0.22 ± 0.06	NR	AUC_6_: 4.90 ± 1.80	HPLC (detector not reported)
[34]	Canada	5.9 [0.6–19.3]	25 [6.9–85.7]	QID; 4	1	0, 0.25, 0.5, 1, 2, 3, 4	0.25 [0.124–0.352]	NR	C_ss_: 576.5 [399–1153] ng·mL^−1^	HPLC–UV
[35]	Canada	5.8 [0.1–19.9]	20.05 [4–87.9]	QID; NR	1	NR	0.246 [0.108–0.432]	NR	NR	HPLC, LC–MS/MS, GC–MS, and GC–ECD (center-specific)
[29]	Korea	Metabolomic: 12.3 [1.3–22.2]; Retrospective: 9.5 [0.6–22.2]	NR	QD; NR	1	after 3 h i.v. 0, 1, 2, 4	0.336 ± 0.204 for ferritin ≥ 1000 ng·mL^−1^; 0.432 ± 0.216 for ferritin < 1000 ng·mL^−1^	NR	AUC_24_: 20.037 [11.668–31.197]	LC–MS/MS
[36]	Canada	8.5 [0.1–21]	NR	QID; NR	1	0, 2, 2.25, 2.5, 3, 4, 5, 6	BuFlu: 0.17 [0.138–0.462]; Other groups: 0.252 [0.108–0.432]	NR	AUC_6_: 1.58–7.19	HPLC–UV
[37]	USA	0.48 [0.05–0.96]	6.0 ± 2.2	QD; 4	TD/RD 1	TD: after i.v. 3, 3.5, 5, 7; RD: after i.v. 3, 3.5, 5, 8, 24	0.22 ± 0.06	NR	TD AUC_6_: 3.95 ± 1.23; RD AUC_24_: 18.82 ± 4.22	GC–MS
[38]	Canada	6.5 [0.1–19.9]	24.1 [4.3–84.8]	QID; 4	1	NR	0.252 [0.108–0.432]	NR	AUC_6_: 3.27 [1.81–7.19]	HPLC–UV
[39]	USA	7.4 [0.2–18.4]	25.5 [7–68]	BD; 4	1	1, 2, 3, 5, 6, 7, 8	0.228 [95% CI 0.204–0.252];	0.70 [95% CI 0.65–0.73]	AUC_12_: 8.28 [95% CI 7.57–8.97]; AUC_24_: 16.55 [95% CI 15.14–17.95]	GC–MS
[40]	Korea	9.3 [0.9–18.1]	BSA: 1.07 m^2^ [0.48–1.89]	QD; 4	1, 4	after i.v. 0, 1, 2, 4	0.242 [0.104–0.416];	NR	AUC_24_: 16.82 [12.08–31.66] (CV 26.5%)	LC–MS/MS
[41]	USA	7.1 [0–21]	30.6 [2.5–117.8]	QD; 3	TD/RD 1	TD: 3, 3.5, 5, 7; RD: 3, 3.5, 5, 8, 24	TD: BMI < 25%: 0.222 ± 0.036; BMI 25–85%: 0.228 ± 0.06; BMI ≥ 85%: 0.192 ± 0.06 RD: BMI < 25%: 0.24 ± 0.066; BMI 25–85%: 0.228 ± 0.066; BMI ≥ 85%: 0.186 ± 0.066	NR	TD: AUC_6_: 3.76 ± 0.77/3.61 ± 0.866/4.46 ± 1.49 RD: AUC_24_: 15.22 ± 3.27/18.05 ± 3.96/16.87 ± 2.77	GC–MS
[42]	Canada	5.2 [0.4–19.8]	24.1 [6.2–84.5]	QID; 4	1	0, 0.25, 0.5, 1, 2, 3, 4	*GSTM1* null: 113.7 ± 0.2 mL·min^−1^·m^−2^; Not *GSTM1* null: 165.2 ± 16.5 mL·min^−1^·m^−2^	NR	*GSTM1* null: AUC_6_: 3.89 ± 0.30; Not GSTM1 null AUC_6_: 3.11 ± 0.19	HPLC–UV
[43]	USA	≤4 years: 1.6 [0.5–3.8]; >4 years: 10.7 [4.5–16.7]	≤4 years: 9.9 [7.1–17.1]; >4 years: 34.5 [16.6–62.6]	QID; 4	1, 9, 13	0, 0.25, 0.5, 0.75, 1, 2, 3, 4, 5, 6	≤4 years 0.246; >4 years 0.174	≤4 years: 0.68–0.72; >4 years: 0.6	AUC_6_: 4.79	GC–MS
[44]	USA	7.7 [0.06–17.6]	25.6 [2.5–117.8]	QD; 3	TD/RD 1	TD: 3, 3.5, 5, 7; RD: 3, 3.5, 5, 8, 24	≤4 years TD: 0.227 ± 0.0744; RD: 0.233 ± 0.048 >4 years TD: 0.202 ± 0.0504; RD: 0.193 ± 0.0648	NR	<4 years TD AUC_6_: 3.64 [1.80–7.50]; RD AUC_24_: 14.65 [6.20–29.78]; >4 years TD AUC_6_: 3.96 [2.38–8.09]; RD AUC_24_: 16.56 [8.34–22.53]	GC–MS
[45]	USA	8 [0.25–18]	34.5 ± 25.7	QID; 4	1	2, 2.25, 2.5, 3, 4, 5, 6	0.21 ± 0.054; <4 years: 0.246 ± 0.06; >4 years: 0.186 ± 0.042	NR	AUC_6_: 4.49 ± 0.93	GC–MS
[46]	Australia	3.2 [0.2–17.7]	14.0 [10.7–33.2]	QD; 4	1	After i.v. 0, 0.5, 1, 2, 4, 6, 8, 10, 12/ 0, 1, 2, 4	0.23 ± 0.08 (CV 35%)	0.74 ± 0.16 (CV 22%)	AUC_24_: 24.7 ± 9.2 (CV 37%)	GC–ECD
[47]	Canada	3.0 [0.25–16.2]	13.7 [4.8–108.7]	QID; 4	1	0, 0.25, 0.5, 1, 2, 3, 4	0.333 ± 0.188	0.68 ± 0.17	AUC_6_: 2.54 ± 0.56	GC–ECD

Abbreviations: AUC, area under the concentration–time curve; BSA, body surface area; BMI, body mass index; Bu, busulfan; CL, clearance; C_ss_, steady-state concentration; CV, coefficient of variation; GC–ECD, gas chromatography with electron-capture detection; GC–MS, gas chromatography–mass spectrometry; HPLC, high-performance liquid chromatography; HPLC–PDA, high-performance liquid chromatography with photodiode-array detection; HPLC–UV, high-performance liquid chromatography with ultraviolet detection; LC–MS/MS, liquid chromatography–tandem mass spectrometry; NR, not reported; BD, twice daily; QD, once daily; QID, four times daily; RD, therapeutic regimen dose; TD, test dose; TDM, therapeutic drug monitoring; V, volume of distribution. AUC_6_, AUC_12_, and AUC_24_ denote AUC over 6-, 12-, and 24 h dosing intervals, respectively; AUC_cum_ denotes cumulative exposure over 96 h, when reported. AUC values were converted to mg·h·L^−1^ where necessary; C_ss_ is presented in ng·mL^−1^. BuFlu, busulfan–fludarabine conditioning regimen; CI, confidence interval; GSTM1, glutathione S-transferase mu 1; i.v., intravenous; SD, standard deviation. Age, weight, CL, V, and exposure values are presented as mean ± SD or median [range], as reported in the original studies. * Studies are presented in reverse chronological order by publication year.

**Table 2:** Summary of busulfan exposure metrics, estimation methods, and exposure–outcome/reference ranges in pediatric HSCT.

Reference	Cohort Size	Study Population or Clinical Scenario	Dosage Regimen	Exposure Metric	Exposure Estimation Method	Exposure Target/Threshold	Primary Basis/Endpoint
AUC_6_							
FDA Label	NR	NR	QID	AUC_6_	Not applicable (label)	4.62 (3.69–5.54)	Therapeutic window recommended by the FDA label
EMA Label	NR	NR	QID	AUC_6_	Not applicable (label)	3.69–6.16	Range recommended by the EMA label
[89]	63	China; Malignant and non-malignant diseases	QID	AUC_6_	Model-based (PopPK/NLME)	3.90–6.57	This range is associated with better EFS
[90]	455	Multicenter; Malignant and non-malignant diseases	QID	First-dose AUC_6_	Model-based (PopPK/NLME)	≥4.5	Higher 2-year EFS
Cumulative AUC							
[11]	102	Netherlands; Malignant and non-malignant diseases	QD, QID	AUC_cum_	Model-based (Bayesian)	78 (74–82)	This exposure level corresponds to optimal EFS; increased toxicity when combined with melphalan at >74
[10]	674	Multicenter; Malignant and non-malignant diseases	QD, QID	AUC_cum_	Model-based (PopPK/NLME)	78–101	Optimal EFS balancing efficacy and toxicity
[91]	57	USA; Non-malignant diseases	QID, BD, QD	AUC_cum_	Model-based (PopPK/NLME)	75 (70–80)	Sustained high-level donor myeloid chimerism and disease correction; authors proposed a target of 75 (70–80)
[92]	562	Multicenter; IEI/primary immunodeficiency-related patients	QD, QID	AUC_cum_	Model-based (PopPK/NLME)	80 (70–90)	Better EFS and higher donor chimerism; donor chimerism plateaus at about 90; EFS relationship differed by IEI subgroup
[17]	697	Multicenter; Malignant and non-malignant diseases	QID, BD, QD	AUC_cum_	Model-based (PopPK/NLME)	>78	Primarily reflects toxicity boundary; AUC_cum_ > 78 associated with increased VOD/SOS when Bu was the sole alkylating agent
[93]	36	Japan; Malignant and non-malignant diseases	QD, QID	AUC_96_	NR	>80	Higher cumulative Bu AUC is associated with an increased risk of neurological complications
[94]	145	Multicenter; ALL	QD	AUC_cum_	Model-based (PopPK/NLME)	73.3–98.0	Optimal EFS/GRFS; low exposure associated with relapse, high exposure with toxicity/GVHD
[90]	455	Multicenter; Malignant and non-malignant diseases	QID	AUC_cum_	Model-based (PopPK/NLME)	Bu3 ≥ 54; Bu4 70–90	Better 2-year EFS
C_max_							
[16]	293	Multicenter; Malignant and non-malignant diseases	QD, QID	C_max, all_	Model-based (Bayesian)	>1.88	Strongest CART predictor of VOD; proposed toxicity upper boundary
[95]	334	Korea; Malignant and non-malignant diseases	QD, QID	End-of-infusion C_max_	Direct measurement	>4.2696	Liver-function-test elevation; lower C_max_ associated with better OS
C_ss_							
[96]	75	Canada; Malignant and non-malignant diseases	QID	First-dose C_ss_	NCA	>600 ng·mL^−1^ (observational cutoff)	Higher NRM and grade II–IV aGVHD; lower EFS and OS
[34]	36	Canada; Malignant	QID	First-dose C_ss_	NCA	>600 ng·mL^−1^	Lower neutrophil and platelet recovery; higher NRM; lower EFS and OS

Abbreviations: ALL, acute lymphoblastic leukemia; aGVHD, acute graft-versus-host disease; AUC, area under the concentration–time curve; AUC_6_, AUC over a single 6 h dosing interval; AUC_96_, cumulative AUC over 96 h; AUC_cum_, cumulative AUC over the full busulfan treatment course unless otherwise specified; BD, twice daily; Bu, busulfan; Bu3 and Bu4, cumulative busulfan exposure through treatment Days 3 and 4, respectively; CART, classification and regression tree; C_max_, maximum plasma concentration; C_ss_, steady-state concentration; EFS, event-free survival; EMA, European Medicines Agency; FDA, U.S. Food and Drug Administration; GRFS, GVHD-free, relapse-free survival; GVHD, graft-versus-host disease; HSCT, hematopoietic stem cell transplantation; IEI, inborn errors of immunity; NCA, noncompartmental analysis; NLME, nonlinear mixed-effects modeling; NRM, non-relapse mortality; NR, not reported; OS, overall survival; PopPK, population pharmacokinetics; QD, once daily; QID, four times daily; VOD, hepatic veno-occlusive disease; VOD/SOS, hepatic veno-occlusive disease/sinusoidal obstruction syndrome. For consistency, all AUC values were expressed in mg·h·L^−1^, whereas C_max_ values were expressed in mg·L^−1^. Exposure estimation method refers to the approach used to derive the exposure metric analyzed against clinical outcomes. Model-based (Bayesian) denotes individual posterior estimation using measured concentrations, whereas model-based (PopPK/NLME) denotes individual or re-estimated exposure derived using a population PK or nonlinear mixed-effects model. C_ss_ denotes the average steady-state concentration derived from AUC over the dosing interval.

## Data Availability

No new data were created or analyzed in this study. Data sharing is not applicable to this article.

## Abbreviations

The following abbreviations are used in this manuscript:

aGVHD	Acute graft-versus-host disease
AI	Artificial intelligence
ALL	Acute lymphoblastic leukemia
AML	Acute myeloid leukemia
AUC	Area under the concentration–time curve
AUC_6_	AUC over a 6 h dosing interval
AUC_12_	AUC over a 12 h dosing interval
AUC24	AUC over a 24 h dosing interval
AUC_96_	Cumulative area under the concentration–time curve over 96 h
AUC_cum_	Cumulative area under the concentration–time curve
BD	Twice daily
BSA	Body surface area
Bu	Busulfan
CL	Clearance
C_max_	Maximum concentration
C_ss_	Steady-state concentration
CYP	Cytochrome P450
DNA	Deoxyribonucleic acid
EBMT	European Society for Blood and Marrow Transplantation
EFS	Event-free survival
EMA	European Medicines Agency
FDA	United States Food and Drug Administration
FMO	Flavin-containing monooxygenase
GC	Gas chromatography
GRFS	Graft-versus-host disease-free, relapse-free survival
GSG	γ-glutamyl-dehydroalanyl-glycine–glutathione S-conjugate
GSH	Glutathione
GST	Glutathione S-transferase
GSTA1	Glutathione S-transferase alpha 1
GSTA2	Glutathione S-transferase alpha 2
GSTM1	Glutathione S-transferase mu 1
GSTP1	Glutathione S-transferase pi 1
GSTT1	Glutathione S-transferase theta 1
GVHD	Graft-versus-host disease
HPLC	High-performance liquid chromatography
HSCT	Hematopoietic stem cell transplantation
IEI	Inborn errors of immunity
IIV	Interindividual variability
IOV	Inter-occasion variability
LC–MS/MS	Liquid chromatography–tandem mass spectrometry
LSS	Limited sampling strategy
MDS	Myelodysplastic syndrome
MIPD	Model-informed precision dosing
ML	Machine learning
NCA	Noncompartmental analysis
NLME	Nonlinear mixed-effects modeling
NRM	Non-relapse mortality
OFV	Objective function value
OS	Overall survival
PBPK	Physiologically based pharmacokinetic
PK	Pharmacokinetic/pharmacokinetics
PK/PD	Pharmacokinetic/pharmacodynamic
PopPK	Population pharmacokinetic
QD	Once daily
QID	Four times daily
RIC	Reduced-intensity conditioning
RUV	Residual unexplained variability
TBI	Total body irradiation
TDM	Therapeutic drug monitoring
THT	Tetrahydrothiophene
UCBT	Unrelated umbilical cord blood transplantation
VOD/SOS	Hepatic veno-occlusive disease/sinusoidal obstruction syndrome
